# Functional brain rewiring and altered cortical stability in ulcerative colitis

**DOI:** 10.1038/s41380-021-01421-6

**Published:** 2022-01-19

**Authors:** Hao Wang, Jennifer S. Labus, Fiona Griffin, Arpana Gupta, Ravi R. Bhatt, Jenny S. Sauk, Joanna Turkiewicz, Charles N. Bernstein, Jennifer Kornelsen, Emeran A. Mayer

**Affiliations:** 1 G. Oppenheimer Center for Neurobiology of Stress & Resilience, UCLA Vatche and Tamar Manoukian Division of Digestive Diseases, David Geffen School of Medicine at UCLA, Los Angeles, CA 90095-7378 USA; 2 Institute of Fundamental and Frontier Sciences, University of Electronic Science and Technology of China, Chengdu, 611731 P. R. China; 3 Imaging Genetics Center, Mark and Mary Stevens Neuroimaging and Informatics Institute, Keck School Medicine at USC, University of Southern California, 4676 Admiralty Way, Marina Del Rey, CA 90292 USA; 4 University of California, Irvine School of Medicine, Irvine, CA 92697 USA; 5 University of Manitoba IBD Clinical and Research Centre, Department of Internal Medicine, Rady Faculty of Health Sciences, Max Rady College of Medicine, University of Manitoba, Winnipeg, Canada

**Keywords:** Diseases, Neuroscience, Diagnostic markers

## Abstract

Despite recent advances, there is still a major need to better understand the interactions between brain function and chronic gut inflammation and its clinical implications. Alterations in executive function have previously been identified in several chronic inflammatory conditions, including inflammatory bowel diseases. Inflammation-associated brain alterations can be captured by connectome analysis. Here, we used the resting-state fMRI data from 222 participants comprising three groups (ulcerative colitis (UC), irritable bowel syndrome (IBS), and healthy controls (HC), *N* = 74 each) to investigate the alterations in functional brain wiring and cortical stability in UC compared to the two control groups and identify possible correlations of these alterations with clinical parameters. Globally, UC participants showed increased functional connectivity and decreased modularity compared to IBS and HC groups. Regionally, UC showed decreased eigenvector centrality in the executive control network (UC < IBS < HC) and increased eigenvector centrality in the visual network (UC > IBS > HC). UC also showed increased connectivity in dorsal attention, somatomotor network, and visual networks, and these enhanced subnetwork connectivities were able to distinguish UC participants from HCs and IBS with high accuracy. Dynamic functional connectome analysis revealed that UC showed enhanced cortical stability in the medial prefrontal cortex (mPFC), which correlated with severe depression and anxiety-related measures. None of the observed brain changes were correlated with disease duration. Together, these findings are consistent with compromised functioning of networks involved in executive function and sensory integration in UC.

## Introduction

Ulcerative colitis (UC) is an inflammatory bowel disease (IBD) characterized by chronically recurring episodes of inflammation of the colon’s mucosal lining followed by variable periods of remission. Symptoms during flares vary in degree and frequency and include abdominal pain, fatigue, weight loss, diarrhea, and bloody stools [1]. The pathophysiology of UC is incompletely understood, but current disease models are restricted to a primary gut-related mechanism, including an aberrant immune response to shifts in the gut microbiome in genetically prone individuals [2]. In contrast, irritable bowel syndrome (IBS) is a disorder of brain-gut interactions that is characterized by chronically recurring abdominal pain and altered bowel habits in the absence of gastrointestinal (GI) inflammation [3, 4]. Both gut disorders often show comorbid symptoms of anxiety and depression, and symptom flares are often triggered by psychosocial stress, suggesting that both diseases share dysregulation in the brain-gut axis [4–6].

Despite the presence of recurrent GI mucosal inflammation, UC patients consistently report less abdominal pain than IBS, suggesting differences in sensory processing and endogenous pain modulation [7]. Abnormalities of perceptual responses between IBS and UC, along with brain imaging studies documenting discrepancies in pain modulating brain regions, have prompted studies investigating differences in the central processing of chronic visceral pain between the two diseases. Compared to IBS, UC patients in clinical remission show greater corticolimbic inhibition associated with reduced perceptual responses to acute rectal balloon distention [7]. Even though the altered brain responses were observed in response to an acute aversive rectal stimulus, one may speculate that these alterations may also play a role in reduced perception of pain during chronic inflammation. Consistent with these observations, increased functional connectivity in corticolimbic regions involving the bilateral middle frontal gyrus, anterior cingulate cortex (ACC), and the left caudate nucleus has been reported in UC patients with active inflammation [2]. In addition to differences in brain function, morphometric differences in the gray matter have also been reported in UC [8]. UC participants compared to either IBS or HC were found to have a greater thickness in cingulate cortex subregions and primary somatosensory cortex but reduced thickness in the orbitofrontal cortex involved in executive functioning and the posterior insula, the primary interoceptive cortex [8]. Using diffusion tensor imaging (DTI) and graph theory network analyses, we recently reported white matter connectivity alterations primarily in brain regions of the visual and somatosensory networks, which were correlated with clinical symptoms of anxiety and depression in UC patients compared to IBS and healthy control participants [9].

Further support for alterations in brain function in UC comes from several studies which have demonstrated a decline in cognitive function in IBD patients [10–12] and patients with other chronic inflammatory diseases [13–15]. A recent epidemiological study demonstrated a significantly increased risk for developing Alzheimer’s disease (AD) in patients with longstanding UC [11]. The link between chronic peripheral inflammation and brain alterations has been attributed to neuroplastic brain changes secondary to neuroinflammation [14]. Together, these findings suggest that chronically recurring gut inflammation may alter morphological, structural, and functional brain features in patients with UC related to both sensory processing and cognitive function.

In the current study, we hypothesized that a history of recurrent colon inflammation is associated with extensive changes in the functional brain connectome, which may explain previously reported perceptual, emotional [16, 17], and cognitive alterations in UC patients [10–12]. To test this hypothesis, we assessed the static and dynamic functional connectome in largely asymptomatic individuals with a history of intestinal inflammation and compared them to two control groups without a history of colon inflammation, with (IBS) without symptoms (HCs). We aimed to identify alterations in global and local network properties which may explain differences in clinical symptoms.

## Methods and materials

### Participants

A total of 222 right-handed individuals participated in the current study, comprising 74 UC (39 female, median age 30 years, range: 18–60 years; median disease duration 11 years, range: 1–50 years), 74 IBS (39 female, median age = 30.5 years, range: 18–57 years; median disease duration: 12 years, range 1–51, and 74 age- and sex-matched HC participants (39 female, median age = 31 years, range: 18–57 years). Participants were recruited from the University of California Los Angeles (UCLA), the wider Los Angeles community, and the University of Manitoba (UM). IBS and HCs were age and sex-matched to UC. All participants were right-handed. UCLA participants were recruited through advertisements circulated through online social media websites, local newspapers, university, and hospital community list serve and mailing lists, and flyers were posted in the greater Los Angeles area and on the UCLA campus. The UM participants were recruited through the UM IBD Research Registry, a population-based registry of individuals in Manitoba with IBD. Individuals in the Registry agree to be contacted about research initiatives, but participation is voluntary. All participants provided written informed consent before the beginning of the experiment. The MRI imaging data were scanned from July 2010 to April 2018. All procedures complied with the principles of the Declaration of Helsinki, and all UCLA participants were approved by the Institutional Review Board at UCLA’s Office of Protection for Research Participants and UM participants by the University of Manitoba Health Research Ethics Board.

### Exclusion criteria

Exclusion criteria included extreme strenuous exercise (more than 8 h per week of continuous exercises such as marathon runners or triathlon athletes), substance abuse or tobacco dependence (smoked half a package of cigarettes or more daily), current regular use of analgesic drugs (including narcotics, opioids, and α2-δ ligands), abdominal surgery (appendectomies, hysterectomies, or cholecystectomies), active corticosteroid use, claustrophobia, metal implants, medical or neurological conditions, and presence of past or current psychiatric disorders, as determined by the Mini International Neuropsychiatric Interview [18].

### Clinical and psychosocial assessments

Demographic, clinical, and psychosocial assessments, including age, sex, education, and body mass index (BMI) [19], were obtained. UC and IBS participants were administered the Bowel Symptom Questionnaire (BSQ) [20], a validated questionnaire assessing self-reported symptom severity of GI symptoms, bloating, and abdominal pain in the past week on a scale from 0–20. A score of zero denotes no complaints, and the highest score refers to severe symptom experience. Other relevant measures include the age of symptom onset, flare frequency, and how long the patient is usually symptom-free. The *Powell Tuck Index* (PTI) score was used to measure symptom severity in UC participants, with scores increasing with symptom severity. A PTI < 5 was used as a measure of remission [21]. In addition, several measures of self-reported symptom severity were assessed, including the *Abdominal Symptom Intensity and Unpleasantness* (24 h); the *Visceral Sensitivity Index* (VSI) [22], a useful self-report measure of the GI symptom-specific anxiety of patients; the *Perceived Stress Scale* (PSS) [23] is the most widely used psychological tool for measuring stress perception.

Several measures of mood, mental and physical functioning, and attribution framework were assessed. These included the *Pennebaker Inventory of Limbic Languidness* (PILL) questionnaire [24], which was used to measure general sensory perception, including visceral and somatic sensations; The *State-Trait Anxiety Inventory* (STAI) [25], one of the most frequently used measures of anxiety in applied psychology, is a 20-item tool with a wider range of scores from 20–80 and scores > 40 were considered clinical cases (clinical diagnosis of generalized anxiety disorder); The *Hospital Anxiety and Depression Scale* (HADS) [26] was used to assess depression and anxiety in the past week; The *12-item Short-Form Health Survey* (SF12) [27] is a generic health rating scale developed to reproduce the physical component scores (PCS) and mental component scores (MCS) of a longer survey, higher values indicate better health.

### MRI data acquisition, quality control, and preprocessing

All participants were scanned using a 3 T Siemens Magnetom Trio at UCLA and 3 T Siemens Magnetom Verio at UM. A high-resolution T1 structural image was acquired with an MPRAGE sequence (TR: 2200 ms, TE: 3.26 ms, TA: 5 min 12 sec, flip angle: 9˚, slice thickness: 1 mm, 176 slices, 256 × 256 voxel matrix, 1 mm voxel size). A 10 min resting-state functional connectivity (RSFC) scan was also acquired (TR: 2000ms, TE: 28 ms, TA: 10 min 4 sec, flip angle: 77˚, slice thickness: 4 mm, voxel resolution: 3.44 × 3.44 × 4 mm, the field of view: 240 × 240 mm, 300 volumes). During the data acquisition, participants were asked to lie quietly in the scanner with their eyes closed. Preprocessing for all modalities was completed in SPM12 (https://www.fil.ion.ucl.ac.uk/spm/software/spm12/). Functional images were preprocessed by first performing transformation from DICOM into NIFTI, realignment and unwarping the data accounting for motion correction (movement-by-distortion interaction), followed by slice-time correction for interleaved slice acquisition, co-registration of structural and functional images using affine registration, segmentation (bias field correction of SPM ‘s unified segmentation) of the structural image gray matter, white matter and cerebrospinal fluid (CSF), and normalization of the images to the MNI152 template with a 4th-degree b-spline interpolation (2 × 2 × 2 mm). All structural images passed quality control assessment based on compliance with the acquisition protocol, full brain coverage, minimal motion, absence of Gibbs ringing, absence of flow/zipper, and minor atrophy/vascular degeneration. Frame-wise displacement (FD) and mean FD (mFD) were obtained for participant exclusion [28]. Subjects with high levels of motion defined by mFD > 0.55 mm were excluded [29, 30]. There were no differences in mFD or the standard deviation of FD between the three groups (See Supplementary Fig. S1). Functional images were then denoised using the CONN toolbox (https://web.conn-toolbox.org/) in MATLAB R2019a. Principal components analysis, i.e., aCompCor [31] was used to derive multiple nuisance signals from white matter and CSF. Linear regression was applied to remove the effects of noise components from white matter and cerebrospinal fluid (five principal components each), estimated subject-motion parameters, the effect of rest, and root-mean-square (RMS) values. A temporal band-pass filter between 0.01–0.08 Hz after regression was used to minimize the influence of physiological, head motion, and other noise sources. The CONN toolbox uses SPMs Fast-Fourier Transformation for band-pass filtering. The denoised images were used for subsequent network analyses. No smoothing was applied to the data.

### Analysis workflow overview

**Fig. 1:**
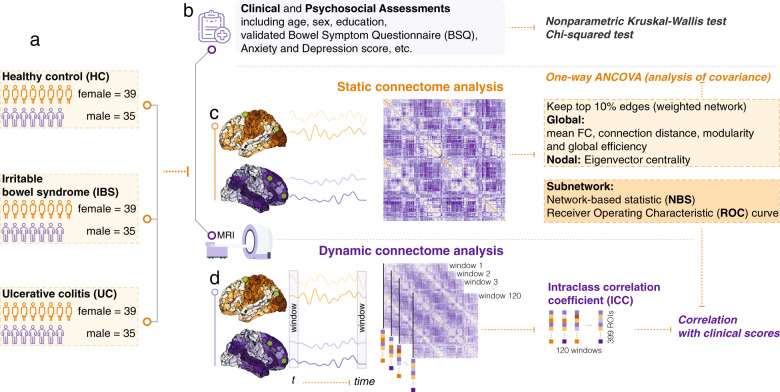
Workflow and overview of analysis in current study. Graph theory was applied to construct and compare functional brain networks. **a** Three age- and sex-matched groups (74 HC, 74 IBS, and 74 UC). **b** Demographic, clinical, and psychosocial assessments were obtained, and statistical analysis was applied to detect the differences among three groups in the measures. **c** Static functional brain network was constructed for each participant, and analysis of covariance (ANCOVA) was applied to examine group differences in the global and regional network topology. Correlation analysis to link the abnormal topological features with each other and clinical scores in UC. The network-based statistic was applied to identify subnetworks within the larger network that show group differences. **d** Dynamic functional analysis was computed using the intraclass correlation coefficient using 120 sliding windows (sliding-window length of 62 repetition times (TR) and 2 TR steps) to assess group differences in cortical stability and investigate the correlation between altered cortical stability and clinical scores in UC.

Figure 1 depicts the analysis pipeline. We applied graph theory to construct and compare functional brain networks among three age- and sex-matched groups (HC, IBS, UC). First, we constructed static functional brain networks for each participant and examined group differences in the global and regional network topology. Next, network-based statistics were applied to identify subnetworks within the larger network that show group differences. We then assessed the utility of using the mean pairwise connectivity from the discriminative brain subnetworks for predicting participant diagnosis. Finally, dynamic functional analysis was used to assess group differences in cortical stability and investigate the correlation between altered cortical stability and clinical scores (Fig. 1).

### Static functional connectome

Regions of interest were defined by the Schaefer (17 networks, 400 parcels, 2 mm) atlas [32]. The connectivity between the regions was calculated by computing the Pearson correlation between average BOLD time series signals across all voxels within each region. This resulted in a 400 × 400 RSFC matrix for each participant, where regions represent the nodes of the network, and the correlations reflect the weighted edges linking the network nodes. This weighted connectivity matrix was thresholded to suppress negative or anti-correlation to zero. Next, each participant’s brain network’s backbone structure was extracted by using Primm’s minimal spanning tree algorithm [33] to keep the graph fully connected, with no isolated nodes. Given the interest in the strongest connections, the matrix weights (i.e., correlations) were inverted, and a maximum spanning tree approach was applied, resulting in a binarized (unweighted) functional matrix comprising 400 nodes and 399 edges. Then, additional edges were added in descending order based on their weight (i.e., correlation) until 10% sparsity was achieved [34]. As a final step, this binarized network matrix is multiplied by the original weighted connectivity network to obtain a weighted connectivity matrix with 10% sparsity, a network with 400 regions, and 7980 connections. These subject-specific matrices were then used to examine group differences in global and regional functional network topology.

#### Computation of global and regional network topology

To identify the differences in global functional connectivity, the mean functional connectivity (i.e., mean value across all edges within the network) was computed for each participant. To determine the differences in brain connectivity distances among the three groups, the edges were categorized as short-range (anatomical distance ≤ 45 mm), middle-range (45 mm < anatomical distance < 75 mm), and long-range (anatomical distance ≥ 75 mm) connections [35]. The proportion of short-, middle-, and long-range connections was then calculated for each participant. Next, the global efficiency and network modularity reflecting the functional integration and segmentation of the network were calculated. Global efficiency denotes the efficiency of information exchange in a parallel system in which all nodes are capable of concurrently exchanging information. Modularity quantifies the capacity of the network to separate into modules or subsystems. High modularity implies dense connections within modules and sparser connections between modules, while decreased modularity indicates a decreased number of edges within modules and a greater number of edges across different modules [36]. The modularity was calculated using the Newman algorithm [37]. At the regional level, we calculated the nodal eigenvector centrality, which measures the quantity and quality of a node’s connections and accounts for both the degree of the given node and the degree of its neighbors. Computation of network metrics was performed using the Brain Connectivity Toolbox [38] (RRID: SCR_004841).

#### Network-based statistic (NBS)

This approach was applied to identify subnetworks or clusters of regions showing differential connections within the larger network that show group differences [39]. Briefly, the network edges (i.e., pairwise connectivity between regions) from the weighted unthresholded connectivity matrix were compared between groups using the two-sample *t*-test resulting in a matrix of *t* statistics. The *t* matrix was then thresholded at *P* < 0.0001, resulting in a set of suprathreshold edges. NBS was then applied to identify the number of supra-thresholded edges that form connected components (i.e., subnetworks in which all pairs of nodes are connected by paths or edges) and determine their size (i.e., number of links). Permutation testing with 10,000 random iterations was used to determine the significance of the identified subnetworks based on their size. Here, *P* adjusted < 0.01 (based on the Permutation test) was used to determine the significance of the subnetworks. For the NBS, the null hypothesis is always rejected at the component level, not at the edge level. Strictly speaking, it is only valid to make inferences about the connected components (subnetwork) as a whole. The NBS analysis was performed by the GRETNA toolbox [40] (RRID: SCR_009487). All statistical analyses were performed in the MATLAB R2019a toolbox. Visualization of brain results was performed using a network surface representation from BrainNet Viewer [41] (RRID: SCR_009446).

#### Predictive modeling

Predictive modeling using logistic regression was applied to further investigate the significant group differences in the subnetworks (UC < HC, UC > HC, UC > IBS). First, a subject-specific subnetwork connectivity index was derived by computing the mean functional connectivity of the identified subnetworks showing group differences. Next logistic regression (MATLAB R2019a, Classification Learner App, the built-in function: *fitglm*) was used to determine whether the derive mean subnetwork connectivity indices could discriminate between the UC and IBS or UC and HC. *Five-fold* cross-validation was used to decide the validity of the model. Specifically, the data was split into five subsets of roughly equal size, randomly chosen. One subset of the data is used to validate/test the model, which is trained using the remaining four subsets. Each subset is validated exactly once, as this process is repeated five times. The predictive accuracy and the area under the receiver operating characteristic (ROC) curve were reported.

### Dynamic functional connectome

The functional connectome is not static but instead evolves over time. To further evaluate the dynamic characteristic of the functional connectome across time, a sliding-window approach was applied to construct a dynamic functional connectome for each participant [42]. A sliding-window length of 62 TR and 2 TR steps were selected, yielding 120-time windows for each subject. For each time window, the connectivity between the regions was calculated by computing the Pearson correlation between average BOLD time series signals across all voxels within each region, yielding 120 subject-specific functional connectivity networks represented in dynamic functional connectivity matrix (i.e., = 400 × 400 × 120) for each participant. Next, for each subject, cortical stability for each region with other regions’ connection across the 120-time windows was computed by the intraclass correlation across (ICC) [43]. See Fig. 1d for details. Cortical stability reflects the variability of functional connectivity or inflexibility, and higher stability indicates lower variability of functional connectivity and lower flexibility.

### Statistical analysis

#### Group differences

All demographic, clinical, and symptom scores were examined with the nonparametric Kruskal–Wallis test. Categorical data were analyzed with a chi-square test. Group differences in global and regional functional network topology derived from static functional connectivity analysis and the ICC value from the dynamic functional connectome analysis were determined using analysis of covariance (ANCOVA) was performed to control the age and sex as confounding factors. NBS analysis was used to identify subnetworks within the larger network that show group differences. As described previously (see section Predictive modeling), logistic regression was used to determine how well the mean functional connectivity of pairwise associations comprising the identified subnetworks could classify/discriminate UC vs. IBS and UC vs. HC. False discovery rate (FDR) correction was performed for multiple comparisons using a 5 percent false discovery rate, i.e., *q* < 0.05.

#### Association between network metrics and clinical scores

Across all groups, partial correlation analysis, controlling age and sex, was performed to detect the association between significantly altered network parameters [i.e., global (connected distance and modularity), regional (eigenvector centrality) metrics, cortical stability, and the subnetwork connectivity index scores based on the NBS analysis. For the UC group, the association of these network parameters with mood and psychosocial measures (i.e., PILL, PSS, VSI, HADS, STAI, SF12) was computed. A 5% FDR correction was applied to determine the significance within each network parameter.

#### Sensitivity analysis

The effect of motion on resting-state fMRI can be attenuated by regression motion parameters but not completely removed [29, 44, 45]. Therefore, to examine the effect of head motion on the robustness of our results, we included mean FD as a covariate in models examining group differences in the distance metrics (i.e., short-, middle-, and long-range connections) and correlations between cortical stability of the mPFC with clinical measures. These analyses indicated the results were robust to the inclusion of this covariate. Details and results from these analyses are present in Supplementary Fig. S2, Supplementary Tables S1–2.

## Results

### Demographic and clinical characteristics

The median disease duration in UC was 11 years (range 1–50 years) and in IBS 12 years (range 1–51). Nineteen IBS participants were constipation-predominant, 32 were diarrhea-predominant, and the remaining 23 participants experienced mixed constipation and diarrhea. In UC, 23% had pancolitis, 5% had subtotal colitis, 32% had left-sided, 21% had rectal or rectosigmoid disease, and in the remaining 19%, the information was not available. Of the UC participants, 14 were on immunosuppressive therapies (10 on thiopurines, 6 on anti-TNF). Of the six participants on anti-TNF, two were also on thiopurines. Thus, a total of 14 participants were on various forms of immunosuppressive therapy). 43 participants were on anti-inflammatory medications (mesalamine or steroids) without additional immunosuppressive meds. The remaining 17 participants were analgesic, herbal meds, or other. All UC participants had a history of steroid use, but none were on steroids at the time of enrollment or during the study. Seventeen UC participants were taking analgesics, compared to seven IBS participants. The median PTI score was three for UC participants (range 0–11). No differences in age or BMI were observed among UC, IBS, and HCs. Kruskal–Wallis tests revealed significant differences in symptoms and clinical scores among the three groups (all *P-values* < 10^−5^). Compared to IBS, UC participants reported lower intensity and unpleasantness of symptoms during the past 24 h, and lower state and trait anxiety (all *P-values* < 0.05), as well as higher (improved) mental component scores on the SF12 (*P* = 0.004). Overall, the UC and IBS groups show more severe symptoms than HC, see Table 1 for details.

**Table 1:** Demographic and clinical characteristics.

Parameter	HC participants (*n* = 74)	IBS participants (*n* = 74)	UC participants (*n* = 74)	*P-value*
Age(y)	31(18–57)	30.5(18–57)	30(18–60)	0.944
Sex				1
No. of men	35	35	35	
No. of women	39	39	39	
BMI	25(19.05–43.59)	23.45(16.29–36.62)	23.80(16.90–37.70)	0.171
Disease duration	NaN	12(1–51)	11(1–50)	0.593
Abdominal symptom intensity 24 h	0(0–5)	9(1–17)	4(0–14)	< 10^−11^*†‡
Abdominal symptom unpleasantness 24 h	0(0–4)	7.5(0–14)	3(0–13)	< 10^−11^*†‡
PILL score	3.5(0–21)	14(3–37)	12(1–32)	< 10^−13^*†
PSS score	9(0–23)	18(1–32)	14(0–29)	< 10^−8^*†
PTI score	NaN	NaN	3(0–11)	
VSI score	0(0–17)	35.5(2–74)	25(0–59)	< 10^−27^*†
HADS anxiety	3(0–13)	7(0–16)	6(1–13)	< 10^−10^*†
HADS depression	0(0–14)	2(0–11)	2(0–13)	< 10^−7^*†
STAI state anxiety	40(34–70)	49.5(35–70)	43.5(34–74)	< 10^−5^*†‡
STAI trait anxiety	42(33–71)	57(36–82)	47(33–70)	< 10^−8^*†‡
SF12 MCS	55.33(38.46–60.92)	44.57(16.16–58.27)	52.70(23.28–61.57)	< 10^−7^*†‡
SF12 PCS	56.42(46.42–61.98)	52.56(28.33–61.40)	52.81(24.46–65.40)	< 10^−8^*†

Table 1 Legend. Unless otherwise indicated, data are median values with ranges (minimum to maximum values) in parentheses. We performed the nonparametric test as values were not normally distributed in at least one group (according to the Lilliefors test). The sex differences were analyzed with the chi-squared test. Other statistical comparisons were performed with Kruskal–Wallis test.

*Post-hoc comparisons indicated significant differences between the HC group and patients with IBS.

^†^Post-hoc comparisons indicated significant differences between the HC group and patients with UC.

^‡^Post-hoc comparisons showed significant differences between patients with UC and those with IBS.

### Global topological organization of the functional connectome

**Fig. 2:**
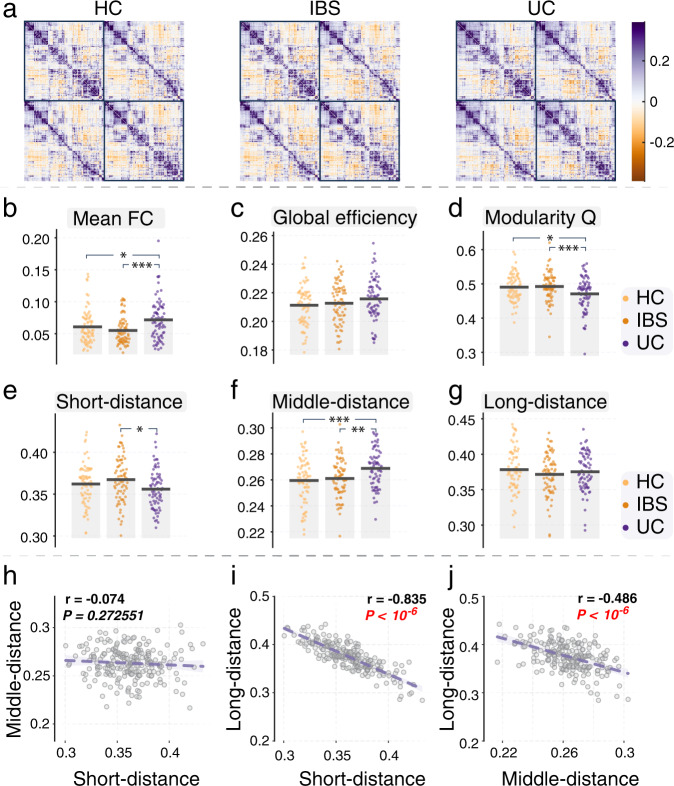
Mean connected matrix, global metrics, and three-type connected distance. **a** Mean connected matrix for HC, IBS, and UC group, separately. **b** Mean functional connectivity for each participant in each group, and the UC group exhibit higher mean FC compared with the HC and IBS group. **c** There is no significant difference among the three groups in global efficiency. **d** The UC groups exhibit decreased modularity compared with the HC and IBS groups. **e** Short-range distance (anatomical distance ≤ 45 mm), **f** Middle-range distance (45 mm < anatomical distance < 75 mm), and **g** Long-range distance (anatomical distance ≥ 75 mm) connections. We observed a lower proportion of short-distance and a higher proportion of middle-distance in UC, compared with the IBS group. ANCOVA and post-hoc tests were performed. *P < 0.05; **P < 0.01; ***P < 0.005. **h** The correlation between the proportion of short-range distance and the proportion of middle-range distance across all participants; **i** The correlation between the proportion of short-range distance and proportion of long-range distance across all participants; **j** The correlation between the proportion of middle-range distance and the proportion of long-range distance across all participants.

ANCOVA and post-hoc tests revealed that UC participants showed higher global functional connectivity strength than both the IBS (*P* = 0.0005, Cohen’s *d* = 0.617) and HC groups (*P* = 0.036, Cohen’s *d* = 0.373), while no difference was observed between HC and IBS participants. No significant group differences were observed for Global Efficiency. Analysis of network modularity showed a significant effect among the three groups (*F* = 5.846, *P* = 0.003), and post-hoc analysis showed UC participants had significantly lower modularity compared with both IBS (*P* = 0.007, Cohen’s *d* = 0.480) and HC (*P* = 0.015, Cohen’s *d* = 0.445) groups (Fig. 2). UC participants exhibited a significantly decreased proportion of short-range connections (*P* = 0.016, Cohen’s *d* = 0.449) and an increased proportion of middle-range connections (*P* = 0.007, Cohen’s *d* = 0.536) compared with IBS, while no significant differences were observed for long-range connections (Fig. 2e-g). See Supplementary Table S3 for details. Correlation analysis across all participants indicated that the proportion of long-range distance was negatively correlated with the proportion of short-range (*r* = −0.835, *P* < 10^−6^) and middle-range distances (*r* = −0.486, *P* < 10^−6^), see Fig. 2h-j.

### Regional topological organization of the functional connectome

**Fig. 3:**
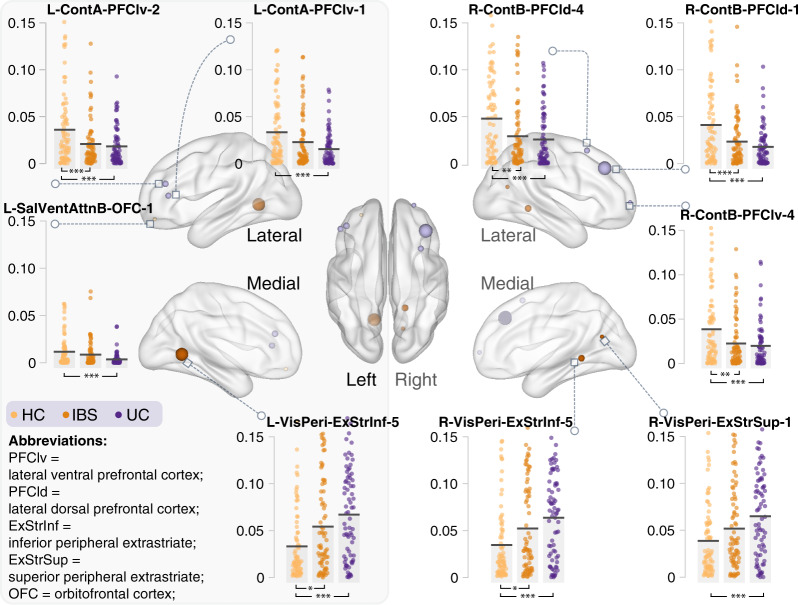
Significant difference in nodal eigenvector centrality among three groups. The UC group shows decreased nodal centrality in the executive network (control and attention) and increased nodal centrality in the visual network. ANCOVA controlling for age and sex and post-hoc test was performed. The horizontal bars indicate mean values. *P < 0.05; **P < 0.01; ***P < 0.005. Cont Control network; VisPeri Visual peripheral; SalVentAttn Salience/ventral attention.

Nine brain regions showed a significant main effect for group on nodal eigenvector centrality (all raw *P-values* < 0.001, FDR-corrected, *q* < 0.05). These regions were primarily located in the salience/ventral attention (orbitofrontal cortex), visual (superior and inferior peripheral extrastriate), and executive control (lateral ventral and lateral dorsal prefrontal cortex) networks (Table 2). Post-hoc comparations revealed that eigenvector centrality showed an increasing pattern (FDR-corrected, *q* < 0.05) from HC to IBS to UC in the visual network (HC < IBS < UC) and a decreasing pattern (HC > IBS > UC) in salience/ventral attention and control networks. No significant correlation between eigenvector centrality and clinical characteristics within these nine brain regions was observed (Fig. 3).

**Table 2:** Nine brain regions show differences in nodal eigenvector centrality.

MNI centroid coordinates			Name of Schaefer-400 parcellation	Subnetworks	ANCOVA				
*x*	*y*	*z*			HC	IBS	UC	*P value*	*F*
−48	35	10	L-ContA-PFClv-1	Control	0.033	0.023	0.015	0.00046†	7.964
−42	38	22	L-ContA-PFClv-2	Control	0.036	0.021	0.018	0.00039*†	8.156
39	33	38	R-ContB-PFCld-1	Control	0.041	0.023	0.018	0.00003*†	11.093
34	15	56	R-ContB-PFCld-4	Control	0.048	0.029	0.026	0.00048*†	7.914
27	59	3	R-ContB-PFClv-4	Control	0.039	0.023	0.020	0.00076*†	7.424
−14	−57	1	L-VisPeri-ExStrInf-5	Visual	0.033	0.054	0.067	0.00003*†	10.776
18	−45	−3	R-VisPeri-ExStrInf-5	Visual	0.035	0.052	0.064	0.00034*†	8.283
16	−66	19	R-VisPeri-ExStrSup-1	Visual	0.039	0.052	0.065	0.00091†	7.230
−27	49	−14	L-SalVentAttnB-OFC-1	Salience/ ventral attention	0.012	0.009	0.004	0.00106†	7.073

Table 2 Legend. Nine regions in the control [Cont], visual [Vis], and salience/ventral attention [Sal/VentAttn] network showed significant differences in nodal eigenvector centrality. For the control network, a pattern of HC > IBS > UC was observed, while for the visual network, the observed pattern was HC < IBS < UC. Also, see Fig. 3 for illustration.

*Post-hoc comparisons indicated significant differences between HC and IBS group.

^†^Post-hoc comparisons indicated significant differences between HC and UC group.

Abbreviations: *PFClv* Lateral ventral prefrontal cortex; *PFCld* Lateral dorsal prefrontal cortex; *ExStrInf* Inferior peripheral extrastriate; *ExStrSup* Superior peripheral extrastriate; *OFC* Orbitofrontal cortex; *MNI* Montreal Neurological Institute; *UC* Ulcerative colitis; *IBS* Irritable bowel syndrome; *HC* Healthy controls; *L* Left hemisphere; *R* Right hemisphere.

### Subnetworks differences

**Fig. 4:**
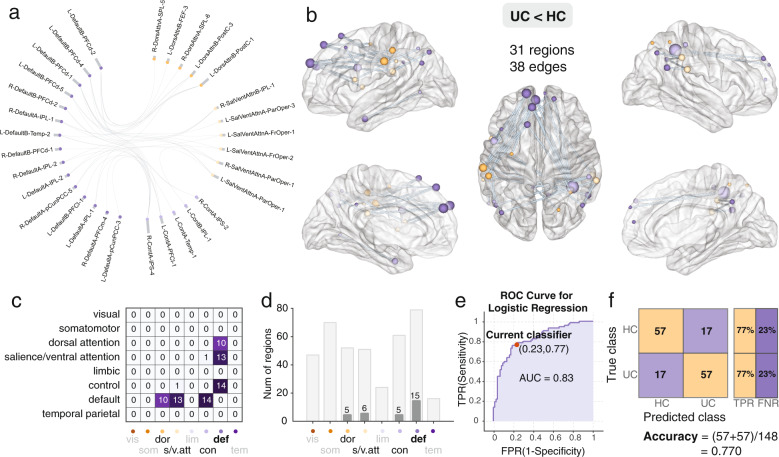
Significant decreased default mode subnetwork in UC group compared with HC group. **a** The circular plot showing the connected brain regions by the network, different networks were colored by different colors and **b** Surface plot showing the spatial location of the brain regions in the decreased connected component revealed by NBS analysis. **c** Number of network-to-network connections of the decreased connected component, there are 10 edges (dorsal attention), 13 edges (salience/ventral attention), and 14 edges (control) with DMN regions. **d** Decreased connected component includes 15 DMN regions, six salience/ventral attention regions, five dorsal attention regions, and five control regions. *Vis: visual; som: somatomotor; dor: dorsal attention; s/v.att: salience/ventral attention; lim: limbic; con: control; def: default; tem: temporal parietal*. **e** The logistic regression reveals good discrimination in distinguishing the UC group from HC group using the decreased connected component, ROC curve (AUC = 0.83), TPR True-positive rate; FPR False-positive rate. **f** The confusion matrix and classification accuracy: 0.77, TPR True-positive rate; FNR False-negative rate. HC Healthy control; UC Ulcerative colitis.

**Fig. 5:**
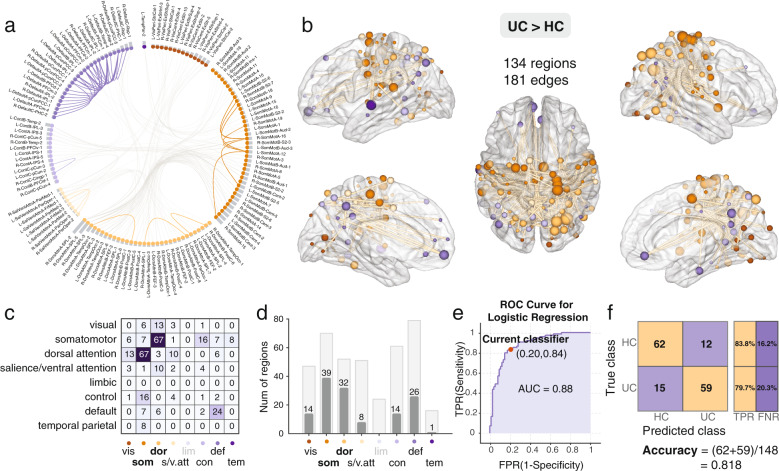
Significant increased connected component in UC group compared with HC group. **a** The circular plot showing the connected brain regions by the network, different networks were colored by different colors and **b** Surface plot of the increased connected component in UC compared to HC, revealed by NBS analysis. **c** Number of network-to-network connections of the increased connected component, there are 67 edges between the somatomotor and dorsal attention regions, there are 24 edges within the DMN regions. **d** Increased connected component mainly includes 39 somatomotor regions and 32 dorsal attention regions. *Vis: visual; som: somatomotor; dor: dorsal attention; s/v.att: salience/ventral attention; lim: limbic; con: control; def: default; tem: temporal parietal*. **e** The logistic regression reveals good performance in distinguishing the UC group from the HC group using the increased connected component, ROC curve (AUC = 0.88), TPR True-positive rate; FPR False-positive rate. **f** The confusion matrix and classification accuracy: 0.818, TPR True-positive rate; FNR False-negative rate. HC Healthy control; UC Ulcerative colitis.

**Fig. 6:**
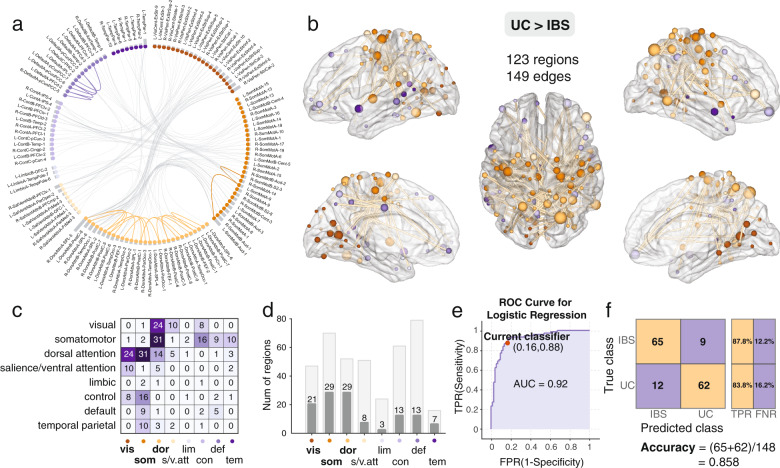
Significant increased connected component in UC group compared with IBS group. **a** The circular plot showing the connected brain regions by network, different networks were colored by different colors and **b** Surface plot of the increased connected component in UC compared to IBS, revealed by NBS analysis. **c** Number of network-to-network connections of the increased connected component, there are 24 edges between the dorsal attention regions and visual regions, there are 31 edges between the dorsal attention regions and somatomotor regions, and there are 16 edges between the somatomotor regions and the control regions. **d** Increased connected component mainly including 29 somatomotor, 29 dorsal attention, and 21 visual regions. *Vis: visual; som: somatomotor; dor: dorsal attention; s/v.att: salience/ventral attention; lim: limbic; con: control; def: default; tem: temporal parietal*. **e** The logistic regression reveals outstanding performance in distinguishing the UC group from IBS group using the increased connected component, ROC curve (AUC = 0.92), TPR True-positive rate; FPR False-positive rate. **f** The confusion matrix and classification accuracy: 0.858, TPR True-positive rate; FNR False-negative rate. IBS Irritable bowel syndrome; UC Ulcerative colitis.

Network-based statistics identified two subnetworks that differed between UC and HC participants. (Fig. 4a-d). One subnetwork (UC < HC) was comprised primarily of default mode network, dorsal attention, salience ventral attention, and control network and was *less* connected (i.e., disconnected) in the UC compared with the HC group. The second subnetwork (UC > HC) was comprised mainly of somatomotor and attention network regions. This ‘somatomotor-dorsal attention’ network was *more* connected in the UC compared to the HC group [*P* < 0.01, corrected] (Fig. 5a-d). For the comparison of UC and IBS, one subnetwork, comprised primarily somatomotor, dorsal attention, and visual networks, was identified and was more connected in UC compared to IBS [*P* < 0.01, corrected] (Fig. 6a-d). No significant subnetwork differences were identified between the IBS and HC groups.

### Average subnetwork connectivity indices predict diagnostic status

Logistic regression indicated that the average functional connectivity of the identified NBS-identified subnetworks distinguished UC participants from HC and UC from IBS. As shown in Fig. 4, the mean functional connectivity of the ‘default mode’ subnetwork discriminated UC from HC with an accuracy of 77% (AUC = 0.83). The average connectivity of the somatomotor-attention subnetwork discriminated the UC group from the HCs with an accuracy of 88% (AUC = 0.88, Fig. 5). Finally, the mean connectivity of the somatomotor-dorsal attention-visual subnetwork predicted UC or IBS status with an accuracy of 86% (AUC = 0.92, Fig. 6).

### Dynamic functional connectivity analysis reveals alterations in cortical stability

ANCOVA analysis revealed a significant effect for the left mPFC (L-DefaultA-PFCm-1) regions (MNI coordinate: *x* = −5, *y* = 55, *z* = −10; *F*_(2217)_ = 10.395, *P* = 4.89 × 10^−5^) and post-hoc tests revealed that the HC group showed lower cortical stability than the IBS (*P* = 0.020, delta = −0.045) and UC groups (*P* = 1.58 × 10^−5^, delta = −0.077). Overall, the mPFC exhibited an increasing trend of cortical stability across three groups (HC < IBS < UC).

### Association among network parameters

Correlation analysis controlling for sex and age indicated that the proportion of short-range connections was positively associated with modularity (*r*_(220)_ = 0.270, *P* = 3 × 10^−5^), with age and sex as covariates. The modularity was negatively correlated with the proportion of middle-range connections (*r*_(220)_ = −0.191, *P* = 0.005) and long-range connections (*r*_(220)_ = −0.130, *P* = 0.053). The latter correlation did not survive FDR correction. Several subnetworks were correlated with the cortical stability of the left mPFC. The UC < HC subnetwork was negatively correlated with the cortical stability of left mPFC (*r*_(72)_ = −0.463, *P* = 0.00004), the enhanced subnetwork UC > HC was positively correlated with the cortical stability of left mPFC (*r*_(72)_ = 0.340, *P* = 0.0003), and the enhanced subnetwork UC > IBS was positively correlated with the cortical stability of left mPFC (*r*_(72)_ = 0.285, *P* = 0.015).

### Clinical correlates of network parameters

**Fig. 7:**
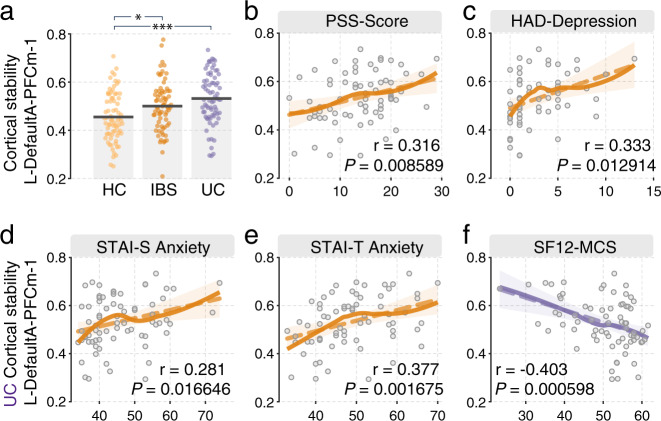
Significant altered cortical stability and correlation with clinical scores. **a** The UC group shows increased cortical stability in left DefaultA-PFCm-1 (mPFC) compared to HC and IBS group. **b** These changes in UC are correlated with PSS-Score (*r* = 0.316), **c** with HAD-Depression (*r* = 0.333), **d** with SATI-S Anxiety (*r* = 0.281), **e** with SATI-T Anxiety (*r* = 0.377), **f** with SF12-MCS (*r* = −0.403). Dashed lines are linear fits, solid lines are local polynomial regression fits (LOESS fits), yellow color indicates positive correlation; purple color indicates negative correlation. PSS Perceived Stress Scale; HAD Hospital Anxiety and Depression Scale; STAI State-Trait Anxiety Inventory; SF12 12-item Short-Form Health Survey; *P < 0.05; **P < 0.01; ***P < 0.005.

Dynamic functional connectome analysis revealed that increases in the cortical stability of left mPFC in UC group were significantly correlated with increased perceived stress (*r*_(72)_ = 0.316, *P* = 0.009), depression (*r*_(72)_ = 0.333, *P* = 0.013), STAI state- (*r*_(72)_ = 0.281, *P* = 0.017) and trait anxiety (*r*_(72)_ = 0.377, *P* = 0.002), and lower scores on the SF-12 mental component (*r*_(72)_ = −0.403, *P* = 6 × 10^−4^). All *p*-values survived after FDR-corrected (*q* < 0.05), see Fig. 7 for details.

For UC participants, no statistically significant correlations between disease duration and mean functional connectivity (FC), global efficiency, and modularity Q were observed at the global level or with the nine nodal eigenvector centrality values at the nodal level. In addition, no significant correlations between disease duration were identified for the left DefaultA-PFCm-1 (mPFC) cortical stability (results show in Supplementary Fig. S3). In addition, we performed the same analysis for IBS participants, which also did not reveal any significant correlations at the global, nodal, and cortical stability levels (shown in Supplementary Fig. S4).

To determine possible differences in *medication usage* for brain findings, we performed an ANCOVA, controlling for age and sex, to compare the differences in brain cortical network changes among these three treatment groups (immunosuppressive, anti-inflammatory, and other), including global and nodal properties, and cortical stability. Statistically significant group differences (*F*_(269)_ = 5.578, *P* = 0.006) were found for global efficiency. As shown in Supplementary Fig. S5A, *post-hoc* tests revealed that the anti-inflammatory group had lower global efficiency when compared both with the immunosuppressive group (*P* = 0.038) and with the group neither on immunosuppressive nor anti-inflammatory medications (*P* = 0.015). No statistically significant differences in mean FC and modularity (Supplementary Fig. S5B-C) were found. For the nodal level and cortical stability, no statistically significant differences were observed (all *P* values > 0.05).

Results from correlation analysis between anxiety and depression scores and other network parameters (including the global and regional topological organization, connectivity distance, and subnetwork connectivity index) for the UC group did not survive after FDR correction (results shown in Supplementary Materials).

## Discussion

This study aimed to test the general hypothesis that a longstanding history of recurrent gut inflammation is associated with extensive changes in the functional brain connectome affecting sensory, emotional, and cognitive function. Confirming our hypothesis, we found converging evidence from several different analyses of alterations in the functional networks linked to executive functioning, including the control network involved in high-level cognitive processing and the dorsal attention and salience/ventral attention involved in goal-directed executive control processes and salience evaluations [46]. These changes, which were not related to disease duration, included greater global resting-state FC, lowered modularity, reduction of short-range brain coupled with increased middle-range connections, and decreased eigenvector centrality in executive control regions. Additionally, network-based statistics revealed differences in default mode network and somatomotor-dorsal attention subnetworks in UC compared to HC, and a somatomotor-dorsal attention-visual subnetwork in UC compared to IBS. These findings expand our knowledge about the effect of chronic systemic inflammation on the brain and cognitive function, in particular regions of the executive control and somatomotor networks [47–49].

Greater global functional connectivity. UC participants exhibited higher global functional connectivity strength compared to both control groups, consistent with altered whole-brain network structures found in chronic pain, several neurological diseases, and disorders associated with abnormal sensory processing of pain, information processing, and transmission between brain networks [50–52].

UC participants show alteration in functional brain wiring and modularity. Functional brain rewiring refers to the reconfiguration or plasticity of the brain on a global scale in response to a disease state. Previous brain mapping studies have suggested abnormal brain wiring is associated with high-cost brain components in neurological disorders, like mild cognitive impairment [35] and global network reconfiguration as a result of a disease’s state [53]. In comparison to IBS and HC groups, UC participants exhibited reduced short-range connections and increased middle-range connections. Neural connections in the brain impose metabolic energy costs such as wiring and oxidative stress, with long distance connections requiring more energy to sustain and higher metabolically expensive networks more susceptible to disruption [54]. Our findings suggest that the functional brain wiring of UC participants is less efficient, implying that it may require a higher metabolic cost compared to the two control groups. Moreover, we observed that alterations in short- and middle-range connections were associated with increased anxiety scores, suggesting a possible link between alterations in functional brain wiring in UC with psychological symptoms.

The brain’s capacity to separate into subnetworks is represented by modularity. Modularity quantifies the extent of subnetwork (or module) division and is an index of cognitive flexibility and neural plasticity [55]. Modular network organization is disrupted in both healthy older individuals and individuals with executive functioning deficits, including cognitive control, attention, and working memory deficits [56–62]. In the current study, UC participants showed significantly lower modularity compared to IBS and HCs. Furthermore, lower modularity was associated with the proportion of short and middle-range connections, suggesting the lower modularity in UC may be driven by the observed differences in functional wiring distances. Together with the link between altered connectivity distance and anxiety, these results suggest that alterations in the proportion of connectivity distance and modularity are associated and may underlie anxiety symptoms in UC.

Reduction in eigenvector centrality in the executive control network regions. To further explore the regional changes among the three groups, we examined the degree of intraregional brain activity between UC, IBS, and HC groups. Brain regions that are more trafficked are identified as nodes with higher values of centrality, while a reduction in the share of network traffic in a particular brain region is indicated by a lowered eigenvector centrality [63]. Assessing the relative connectedness of brain regions accounts for the influence of the measured node on neighboring regions, a property referred to as eigenvector centrality [64]. UC participants showed decreased eigenvector centrality in five regions within the executive control network (HC > IBS > UC), suggesting less traffic within the executive control network. These alterations could represent functional topographical evidence of the reduction in executive functioning, which has been reported in patients with IBD [2], chronic pain [65], and other chronic inflammatory conditions [49, 66]

Reduced flexibility of mPFC. The human ability to flexibly alternate between tasks is a central component of cognitive control. Cognitive flexibility [67, 68] has been identified as a complex interaction of several mechanisms integrating task demands and sensorimotor aspects and is believed to originate partly from alterations in networks involving both medial and lateral PFC [69–71]. Cognitive flexibility is anticorrelated with cortical stability, a measure of FC variability. In this study, the UC group exhibited greater cortical stability (e.g., lower flexibility) in the medial PFC, and this was significantly associated with a lower modularity index in this sample. Medial PFC plays a crucial inhibitory role in affective brain regions, and mPFC dysfunction has been identified in affective disorders [72–74]. This is consistent with the current findings that showed that the lower cortical flexibility of the mPFC in UC was significantly correlated with increased *State-Trait Anxiety Inventory* (STAI) state- and trait anxiety, depression, and a worse mental component score in UC.

### Subnetwork differences in UC

Compared to HCs, the UC group showed *reduced* connection strength between a default mode subnetwork with the dorsal attention, salience/ventral attention, executive control networks, a finding also observed in patients with mild cognitive impairment [35], AD patients [75], and cognitively normal elderly with elevated brain amyloid [76]. In early AD, resting-state functional connectivity of the precuneus (a key region of the default mode network) with the visual cortex was significantly increased [77, 78]. In addition to findings in AD, disruptions in the functioning of the default mode network have previously been reported in patients with chronic pain conditions, including chronic back pain, complex regional pain syndrome, and knee osteoarthritis, all of which show decreased connectivity of the PFC to subnetworks of the DMN [79]. In IBD, changes in the default mode network have been implicated in the altered processing of homeostatic stimuli and emotional stimuli [80–82].

UC participants also showed *enhanced* connections between the dorsal attention and somatomotor subnetwork, compared to HCs, and these enhanced subnetwork connections (UC > HC and UC > IBS) were associated with the reduced flexibility of left mPFC in the UC group. The link between the lower cortical flexibility of left mPFC and enhanced subnetwork connectivity (dorsal attention and somatomotor) may reflect a reduced ability to disengage from hypervigilance to somatic sensations in UC compared to IBS and HC. Converging evidence suggests that the enhanced FC between attentional and somatomotor networks observed in the current study in UC participants has also been associated with aging [83].

The current study demonstrates enhanced FC among somatomotor, dorsal attention, and visual networks. UC participants also displayed a greater eigenvector centrality in three regions within the visual network (UC > IBS > HC), representing enhanced regional interactions in the visual network and cortical areas. This finding is consistent with previous studies showing structural and functional alterations in the visual network in individuals with chronic inflammation [84–86], including IBD [87], and in patients with chronic pain symptoms, including migraine and chronic low back pain [85]. In addition, an increased visual cortical activation was found in a cognitive decline group in a task fMRI study [88]. Even though the precise role of alterations in the visual network in chronic pain, inflammation, and cognitive function remains to be determined, currently available data suggest it is part of an integrative convergence zone receiving and processing multisensory input, which may play a role in multisensory hypersensitivity. To our knowledge, this is the first study that demonstrates the greater centrality of regions within the visual network of UC participants in comparison to IBS.

The clinical diagnosis was predicted by group differences between the average FC of subnetworks with high accuracy. These findings suggest that alterations of average FC of regional subnetworks may help differentiate between inflammatory and functional intestinal diseases which present with similar clinical symptoms.

### Limitations

This study was a cross-sectional analysis comparing brain features in participants with a chronic history of recurring colon inflammation with two non-inflammatory control groups. It was not aimed at identifying brain changes associated with acute gut inflammation, nor can it answer questions about causality between chronic gut inflammation and brain changes consistent with altered cognitive function and sensory modulation. Even if the observed brain changes are associated with inflammation, it’s not possible to determine whether these changes reflect maladaptive or compensatory processes in UC. The goal of the study was to test the hypothesis that a longstanding intermittent exposure of the brain to inflammatory mediators from recurrent colon inflammation results in distinct functional brain changes which may be related to previously reported sensory, cognitive, and emotional clinical features. Because of our specific hypothesis, we did not assess the presence of mucosal inflammation through analysis of fecal calprotectin levels in the UC participants. We cannot answer the question of how long colon inflammation has to be present before brain changes are detectable. However, correlational analyses between observed brain alterations and disease duration did not reveal statistically significant correlations. UC participants were on different medications at the time of the study, 14 taking immunosuppressive and 43 anti-inflammatory medications. It is conceivable that the immunosuppressive group (a) represented a more severe spectrum of disease with greater brain changes, (b) that the more aggressive therapy resulted in fewer brain changes, or (c) that the immunosuppressive medications themselves contributed to the observed brain changes. While global efficiency, a measure of enhanced capacity for brain communication was higher in the immunosuppressive group, it was similar to the participants without specific therapies, and there was no difference in disease severity when compared to the other groups. These findings argue against the first hypothesis but do not allow us to differentiate between the others. Another limitation was the lack of visual or cognitive assessments performed in our subjects, as the main hypotheses were disease-related brain alterations in sensory and emotional brain networks. Even though UC participants reported a median disease duration of 11 years, based on PTI and BSQ scores, UC participants were only mildly symptomatic at the time of the study, and we were unable to perform correlational analyses of brain changes with abdominal symptoms.

## Conclusions and Clinical Implications

Even though we were not able to correlate brain findings with potential clinical implications, our findings are most consistent with functional brain alterations related to executive functioning and sensory integration. We found the cortical stability of left mPFC was correlated with clinically relevant measures of anxiety and depression. The identification of reorganization of the default mode, somatomotor/dorsal attention, and visual networks identified in UC may have implications for interventions [89] aimed at reducing the risk of development in cognitive decline in vulnerable individuals.

## Supplementary information

Supplemental Material

## Data Availability

The fMRI data were preprocessed by SPM12 and CONN toolbox (https://web.conn-toolbox.org/); Graph theory analysis were performed by Brain Connectivity Toolbox (https://sites.google.com/site/bctnet/). The NBS analysis was performed by GRETNA toolbox (https://www.nitrc.org/projects/gretna/). Visualization of brain results was performed by BrainNet Viewer (https://www.nitrc.org/projects/bnv/). Custom codes are not currently provided or deposited in a public repository. Analysis code is available from the authors upon request.
