# Supplementary material for: Functional brain rewiring and altered cortical stability in ulcerative colitis
Source: Mol Psychiatry. 2022 Jan 19;27(3):1792–804. doi: 10.1038/s41380-021-01421-6 (PMC9095465; doi:10.1038/s41380-021-01421-6)
Supplement: Supplementary file 1 — Supplemental Material [file 41380_2021_1421_MOESM1_ESM.docx]

***­Supplementary materials***

**Functional Brain Rewiring and Altered Cortical Stability in Ulcerative Colitis**

Hao Wang^1,2^, Jennifer S. Labus^1^, Fiona Griffin^1^, Arpana Gupta^1^, Ravi R. Bhatt^3^, Jenny S. Sauk^1^, Joanna Turkiewicz^4^, Charles N. Bernstein^5^, Jennifer Kornelsen^5^, Emeran A. Mayer^1*^.

**Effect of head motion**

As shown in **Figure S1**, we performed ANOVA to detect the differences in mFD between the three groups. The results show no significant differences between the three groups (F_(2, 219)_ = 0.09, *P* = 0.918). Furthermore, we also calculated the standard deviation of FD for each participant. Again, there were no significant differences in the standard deviation of FD (sdFD) among the three groups (F_(2, 219)_ = 0.26, *P* = 0.769).


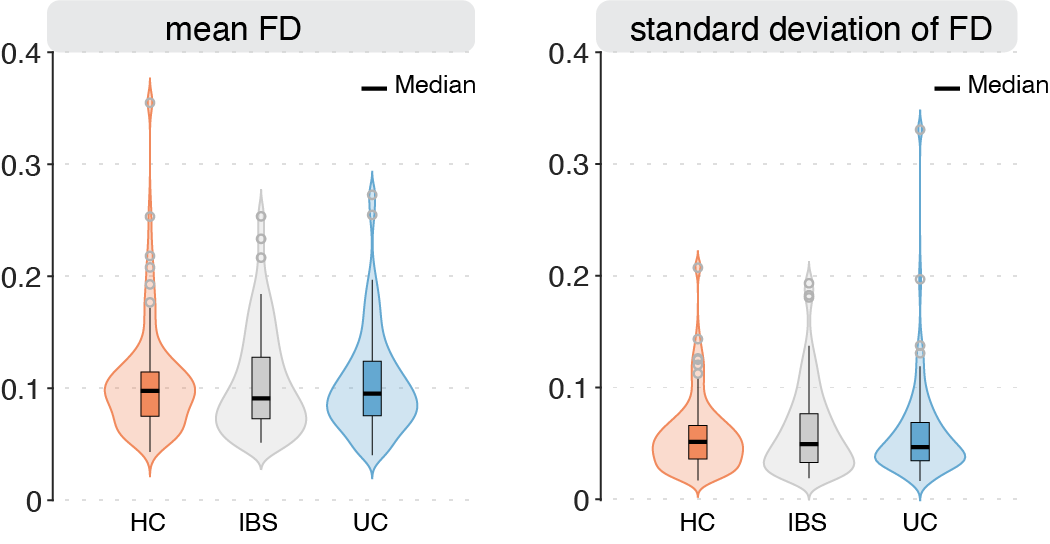


**Figure S1**. Differences in mFD and sdFD. No group differences are observed among the three group. mFD, mean FD; sdFD, standard deviation of FD; HC, healthy control; IBS, irritable bowel syndrome; UC, ulcerative colitis.

**Robustness of our main results against head motion**

To evaluate the robustness of our main results for group difference in the three types of connections, we added mFD as a regressor to the statistical model (i.e., ANCOVA controlling age, sex, and mFD). Adding mFD as a covariate did not alter the results (see **Table S1**)

**Table S1** | Differences among the three groups in three types of connections

|  | Control age & sex | | Control age, sex, & mFD | |
| --- | --- | --- | --- | --- |
|  | F | *P* | F | *P* |
| SHORT | 3.9454 | **0.0207** | 4.0426 | **0.0189** |
| MIDDLE | 7.9066 | **4.85E-04** | 7.9018 | **4.88E-04** |
| LONG | 0.9873 | 0.3742 | 1.0218 | 0.3617 |

Additionally, we examined the effects of controlling for mFD on the associations between the cortical stability of *L-mPFC* and clinical scores (**Table S2**). Again, it did not alter the reported results in the main manuscript.

**Table S2** | Partial correlation between the cortical stability of *L-mPFC* and clinical scores

|  | control age & sex | | control age, sex, & mFD | |
| --- | --- | --- | --- | --- |
|  | r | *P* | r | *P* |
| PILL-Score | 0.212 | 0.124591 | 0.214 | 0.123848 |
| PSS-Score | **0.316** | **0.008589** | **0.305** | **0.011946** |
| VSI-Score | 0.018 | 0.886578 | 0.007 | 0.957023 |
| HAD-Anxiety | 0.197 | 0.149709 | 0.197 | 0.152867 |
| HAD-Depression | **0.333** | **0.012914** | **0.339** | **0.012051** |
| STAI-S Anxiety | **0.281** | **0.016646** | **0.286** | **0.015773** |
| STAI-T Anxiety | **0.377** | **0.001675** | **0.389** | **0.001234** |
| SF12-MCS | **-0.403** | **0.000598** | **-0.396** | **0.000817** |
| SF12-PCS | -0.115 | 0.348751 | -0.099 | 0.419858 |

**Correlation between mFD and three types of connection**

Association between mFD with three types of distance metrics (i.e., long-, mid-, short-range connections). After FDR correction, a significant positive correlation was observed between the mFD and proportions of short-range distance connection in the HC (r = 0.290, *P* = 0.012) and IBS groups (r = 0.284, *P* = 0.014), and a significant negative correlation (r = -0.298, *P* = 0.010) was detected between the mFD and proportions of middle-range distance connection in the HC group.


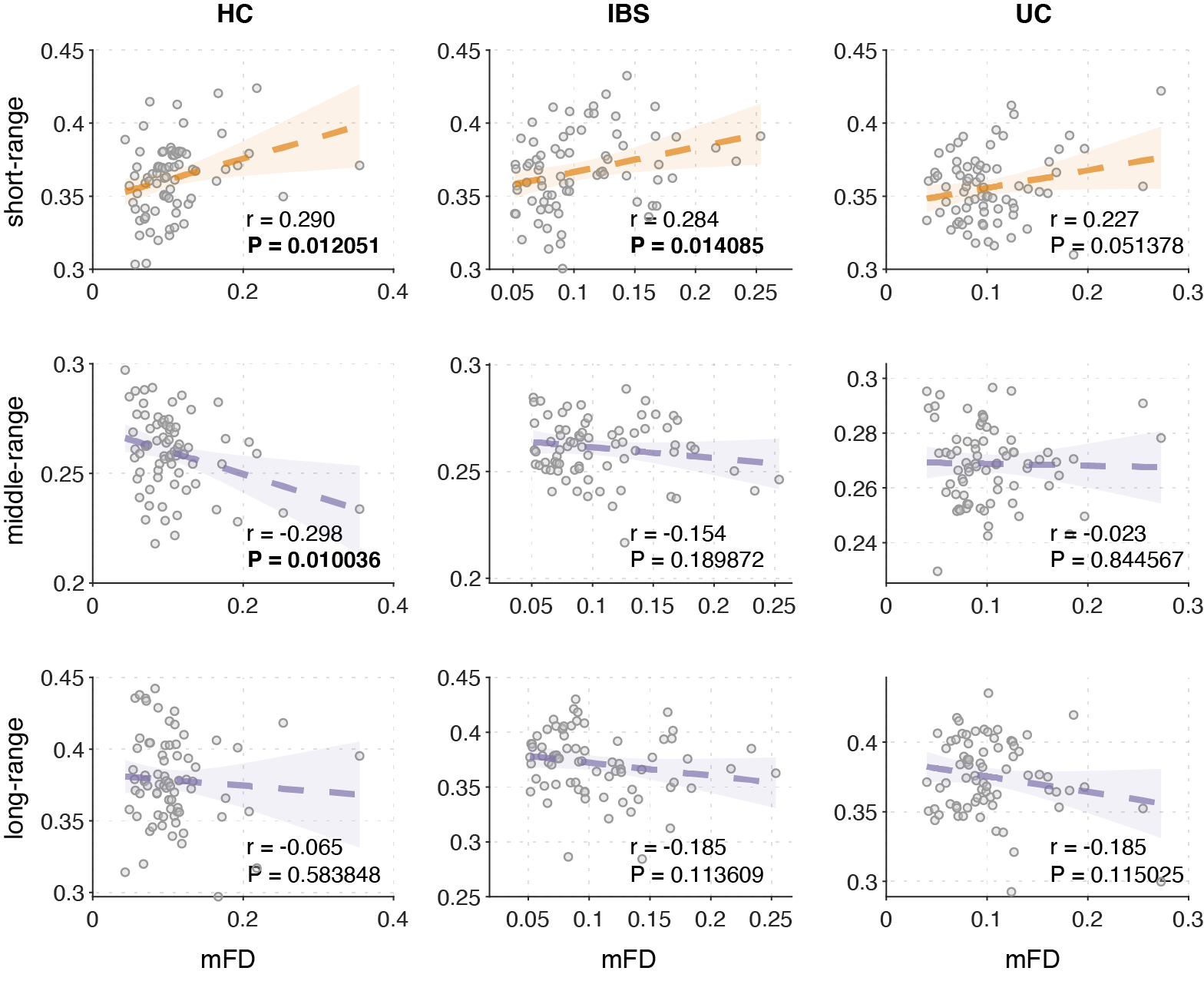


**Figure S2**. Correlation between mFD and three types of connections. We observe that the mFD has a significant effect on *short-range* connections in the HC and IBS group (FDR correction). mFD, mean FD; HC, healthy control; IBS, irritable bowel syndrome; UC, ulcerative colitis.

**Table S3.** **Differences of global topological metrics among three participant groups**

|  | HC | IBS | UC | HC vs IBS | | UC vs HC | | UC vs IBS | |
| --- | --- | --- | --- | --- | --- | --- | --- | --- | --- |
| mean FC | 0.061  (0.02) | 0.055  (0.02) | 0.072  (0.03) | *P = 0.4103* | *d = 0.2320* | ***P = 0.0357*** | *d = 0.3727* | ***P = 0.0005*** | *d = 0.6172* |
| global efficiency | 0.211  (0.01) | 0.213  (0.01) | 0.216  (0.01) | *P = 0.8106* | *d = 0.1030* | *P = 0.1413* | *d = 0.3093* | *P = 0.4101* | *d = 0.2074* |
| modularity | 0.490  (0.04) | 0.492  (0.04) | 0.471  (0.05) | *P = 0.9665* | *d = 0.0443* | ***P = 0.0146*** | *d = 0.4445* | ***P = 0.0067*** | *d = 0.4796* |
| short-distance | 0.362  (0.02) | 0.367  (0.03) | 0.356  (0.02) | *P = 0.4191* | *d = 0.2004* | *P = 0.2928* | *d = 0.2564* | ***P = 0.0162*** | *d = 0.4492* |
| midle-distance | 0.260  (0.02) | 0.261  (0.01) | 0.269  (0.01) | *P = 0.8232* | *d = 0.0938* | ***P = 0.0008*** | *d = 0.5852* | ***P = 0.0066*** | *d = 0.5360* |
| long-distance | 0.378  (0.03) | 0.372  (0.03) | 0.375  (0.03) | *P = 0.3375* | *d = 0.2240* | *P = 0.7906* | *d = 0.1076* | *P = 0.7316* | *d = 0.1278* |

Means and standard deviations are reported. *P* indicates the significant *P*-value; *d* indicates the Cohen’s d value. HC: healthy control; IBS: irritable bowel syndrome; UC: Ulcerative colitis.

**Clinical correlates of network parameters.**

Global and regional topological organization**.** For the UC group, no significant correlations were observed between the mean functional connectivity with depression or anxiety, and lower modularity was associated with higher STAI trait anxiety scores (r_(72)_ = -0.267, *P* = 0.029). *However, this result did not survive after the FDR correction*.

Connectivity distance**.** For UC group, increase in the proportion of short-range connection positively correlated with higher scores on the mental component of the SF-12 (r_(72)_ = 0.254, *P* = 0.035) and reduced STAI trait anxiety (r_(72)_ = -0.261, *P* = 0.033). However, increases in the proportion of middle-range connections were associated with lower scores on the SF-12 mental component (r_(72)_ = -0.304, *P* = 0.010). *None of these results survived after FDR correction*.

Subnetwork connectivity index**.** Correlation analysis reveals the mean connectivity strength in the component (UC > HC) positively correlated with HAD-Depression scores in UC group (r = 0.267, *P* = 0.048, uncorrected). We observed positive correlations, in the UC group, between the mean connectivity strength in the component (UC > IBS) and HAD-Anxiety (r = 0.268, *P* = 0.048, uncorrected) and HAD-Depression (r = 0.354, *P* = 0.008, uncorrected) and STAI-T Anxiety (r = 0.279, *P* = 0.022, uncorrected). We did not observe any significant correlation between the mean connectivity strength in component (UC < HC) and clinical scores.

**Correlation between disease duration and altered brain properties in UC participants**


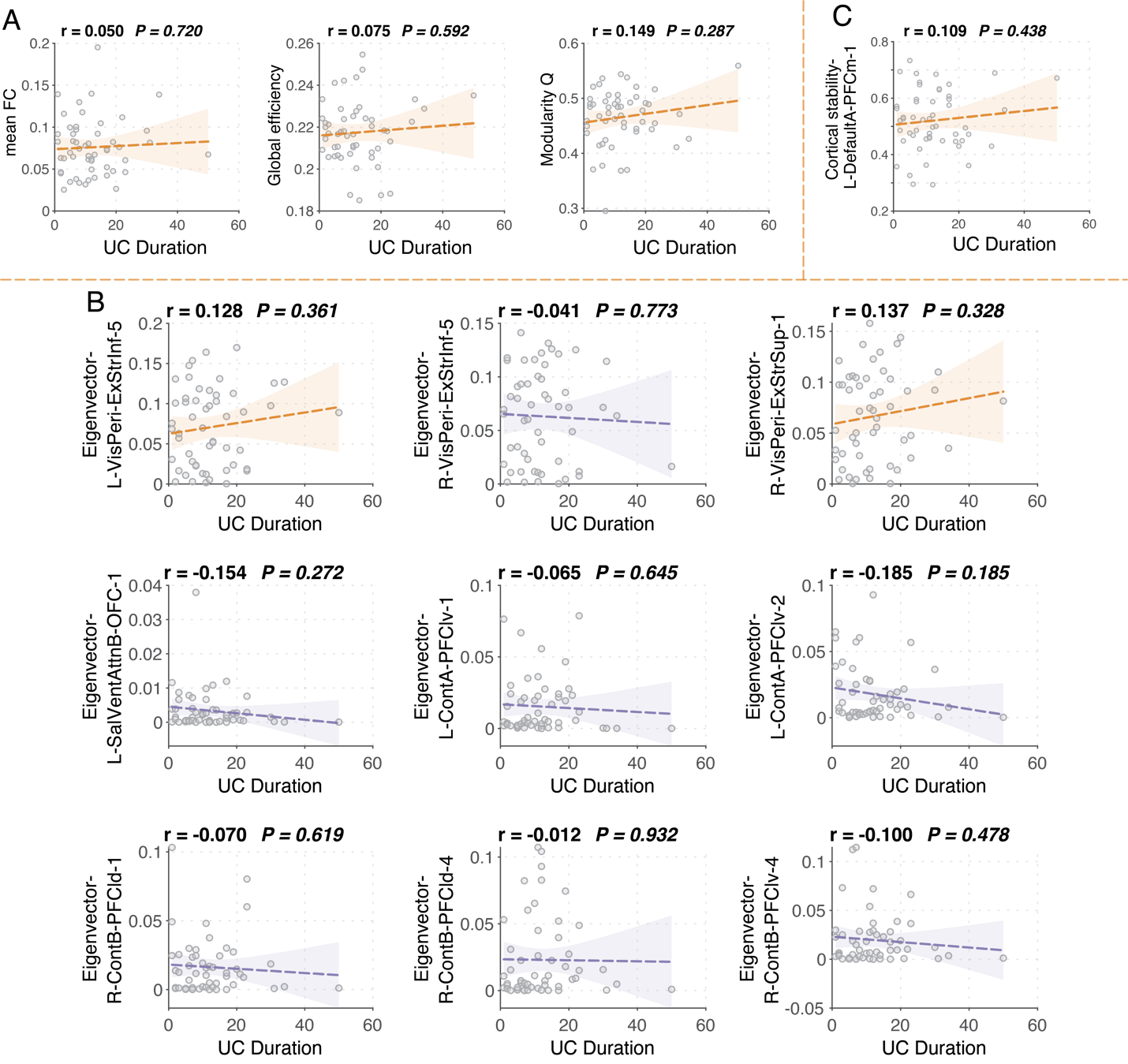


**Figure S3**. Correlations of disease duration with global properties (A), with nodal properties (B) and with cortical stability (C). None of the correlations reached statistical significance.

**Correlation between disease duration and altered brain properties of IBS**

Possible correlations between disease duration and functional brain networks of IBS group were analyzed at three levels (global, nodal and cortical flexibility). *At the global level*, no statistically significant correlations between mean functional connectivity (FC), global efficiency, and modularity Q with disease duration were identified (**Figure S4A**). *At the nodal level*, no statistical correlations between nine nodal eigenvector centrality values and disease duration were identified (**Figure S4B**). *For cortical stability*, no statistically significant correlations of cortical stability of left DefaultA-PFCm-1 (mPFC) with disease duration were identified (**Figure S4C**).


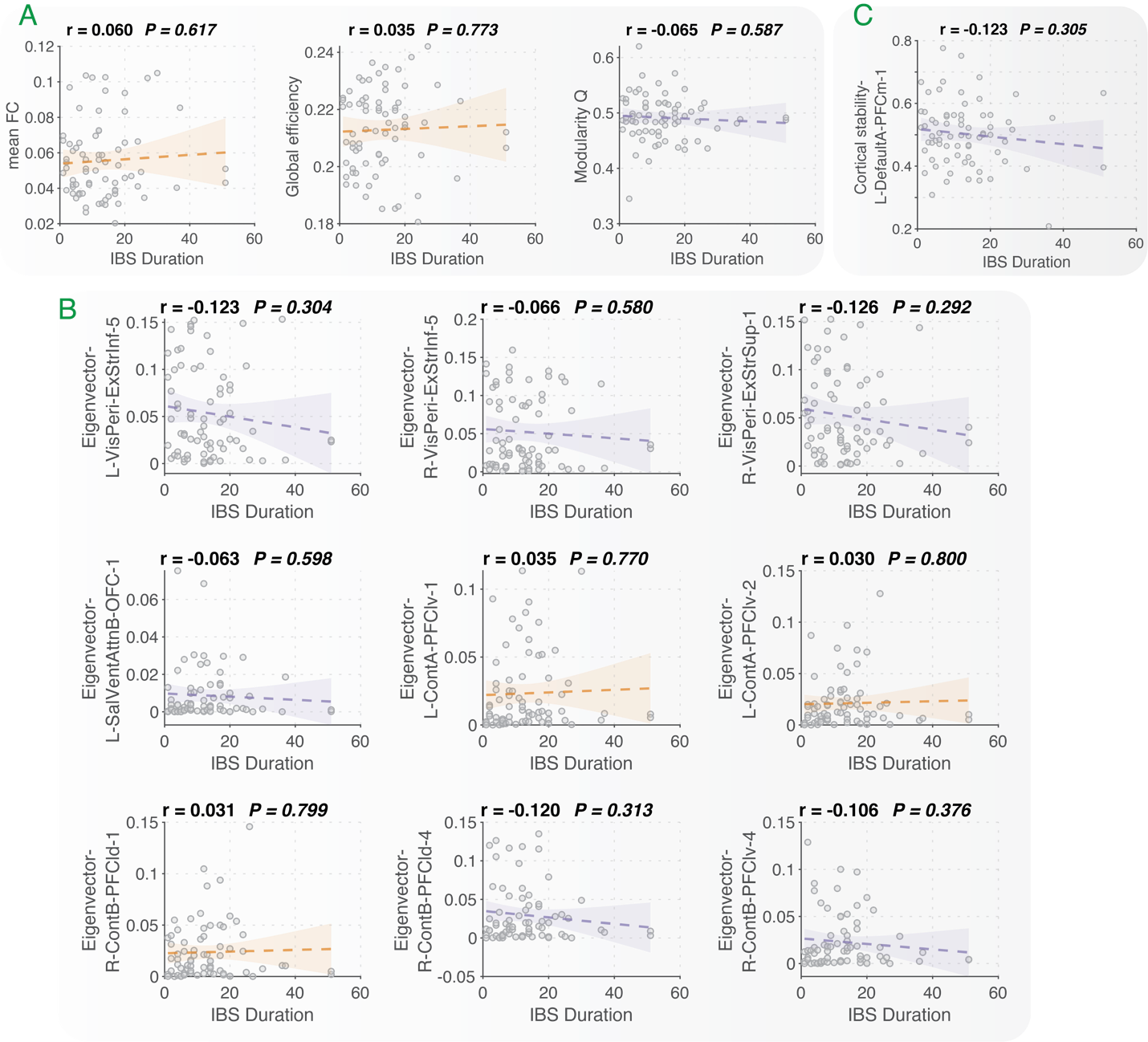


**Figure S4**. Correlation between disease duration and altered brain properties in IBS participants. Correlations of disease duration with global properties (A), with nodal properties (B) and with cortical stability (C).

**Different medication usage on functional brain network in UC participants**

**
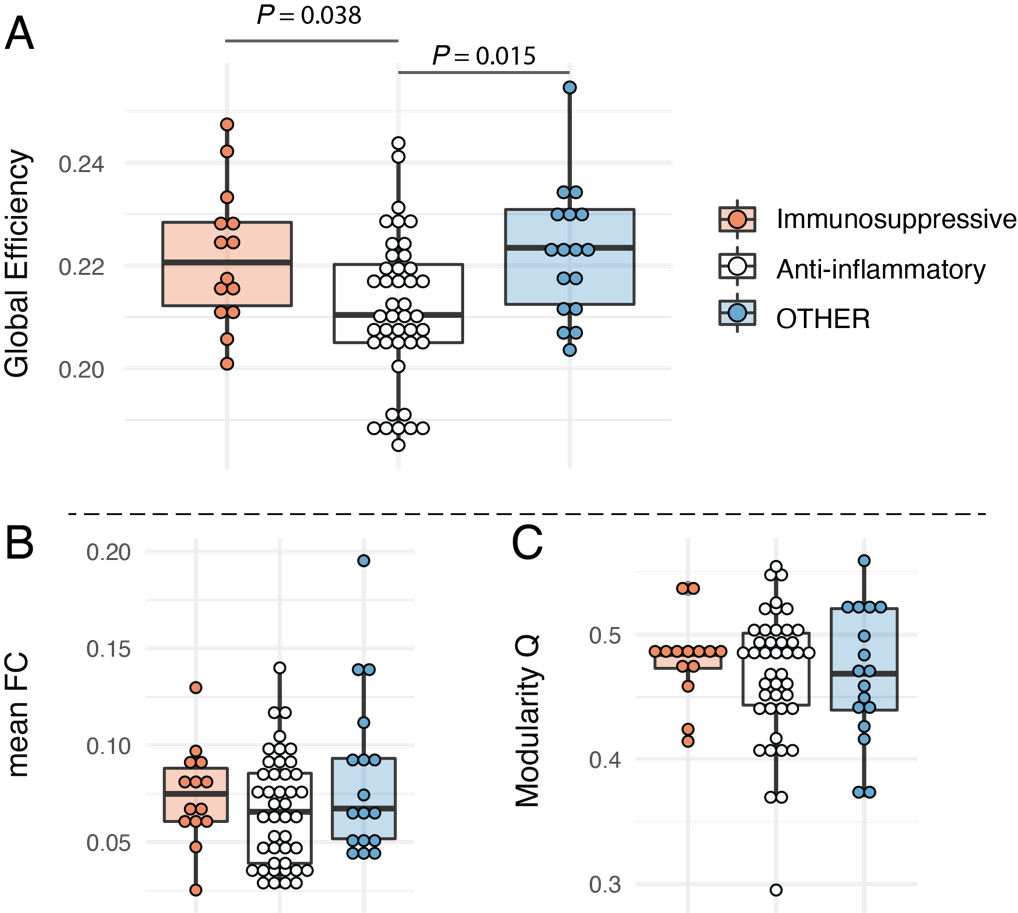
**

**Figure S5**. Effects of different medication usage on functional brain network in UC participants. Significant differences were only observed for *global efficiency* (A), but not for *mean FC* (B) and *modularity Q* (C).
